# A study of correlation between the ect and ehd properties of different aged insulating oil mixtures that can Be an alternative to mineral oil for the insulation and cooling of power transformers

**DOI:** 10.1016/j.heliyon.2022.e12693

**Published:** 2023-01-04

**Authors:** Jilani Rouabeh, Walid Nsibi, Lotfi M’barki, Sameh Khmeilia, Brahim Jallouli

**Affiliations:** a Faculty of Sciences Gafsa, Department of Technological Sectors, Tunisia; b National School of Advanced Sciences and Technologies at Borj Cedria, ENSTAB, University of Carthage, Tunisia; c Electric Transport Base of Gafsa- Tunisia, BTEGF- STEG, Tunisia

**Keywords:** Electro-hydrodynamic phenomenon, Electro-convection, Insulating oils, Oil mixture, Power transformers, Electric field

## Abstract

This article presents the hypothetical steps leading to the damage of power transformers in order to examine whether there is a correlation between the electrostatic properties (ECT) and the electro-hydrodynamic properties (EHD) of insulating oils. Vegetable oils such as, olive oil (OO) and sunflower oil (SO) have been tested as alternative oils for the insulation and cooling of transformers and their blends with naphthenic mineral oil (MO) on the basis of well-defined ratios and for a remarkable aging time, which is an original contribution and insight offered by this paper. The results obtained for the different mixtures with regard to the electrostatic properties and the electro-hydrodynamic properties as well as the physico-chemical properties (power factor “tgδ”, resistivity, conductivity and viscosity) in terms of the aging time were compared with that of the mineral oil. The study of the physico-chemical properties of insulating oils and their mixtures is carried out according to international standards.

## 1 Introduction

Between 1970 and 1985, several cases of breakdown of power transformers took place around the world, most of which were registered in industrially developed countries such as Japan and the United States [1,2]. This breakdown of energy transformers with forced oil flow has been attributed to static electrification and this was noticed from visible discharges and traces of erosion on the insulating paper [3,4].

Since that time, numerous studies have been undertaken not only by academic researchers but also by producers of insulating oils, manufacturers and users of transformers. According to Fleszynski et al., in addition to the phenomenon of static electrification, the electro-hydrodynamic phenomenon that occurs inside the transformer can also play a very important role in the breakdown of these energy devices [5]. Several researchers such as Theodosius et al. have shown that the electro-hydrodynamic phenomenon can play two roles, one positive and the other negative [6]. The positive role of electro-convection is related to the effective electro-hydrodynamic discharge of charges, leading to a reduction in local charge densities and electric field strength. Intensive electro-convection flows - in their negative role -lead to the development of cavitation processes, initiating electric discharges in the oil [[6], [7], [8]]. Today, these phenomena of static electrification and electro-convection of insulating oils therefore concern several researchers around the world [5,16,17].

Over the past twenty years, much research has been carried out to study the phenomena of static electrification and electro-convection in insulating oils [9,11,13,14,18]. According to these advanced studies, the mechanism of static electrification and electro-convection in liquid dielectrics is closely linked to the formation of a double layer of electric charges on the solid/liquid interface [9,18]. These studies have therefore allowed us to assume that the electrical phenomena that occur on the insulating paper and oil interface (inside the transformer) affect both the susceptibility of this oil to static electrification during these flows and its tendency to electro-convulsive motion in an electric field. This supposition led us to develop a scenario concerning the probable stages leading to the damage of the transformers and to formulate hypothesis that posits the existence of a correlation between the electrostatic properties and the electro-hydrodynamic properties of the insulating oils.

The objective of our work is to present the different hypothetical stages of failure of a power transformer (paragraph 2), on the premise of the key role played not only by electrostatic but also by electro-hydrodynamic processes. The importance of the phenomena of static electrification and electro-convection in the oils used for the insulation and cooling of transformers therefore entails the need to study the electrostatic and electro-hydrodynamic properties of these liquid dielectrics. These unconventional properties which, until recently, constituted a very interesting line of research, are characterized by.

- The susceptibility of oils to static electricity (electrostatic properties), already adopted in the literature under the abbreviation ECT (electrostatic charging tendency),
- The tendency of oils to move in an electric field (electro-hydrodynamic properties), known by the abbreviation EHD.

We have known that naphthenic mineral oil has long been the preferred insulating liquid for insulating and cooling power transformers due to the advantages it possesses such as good thermal cooling capacity, good pour point at low temperature in addition to its low cost and wide availability in the market [9,10,12,14,20]. Despite all these advantages, users especially of power transformers have referred to some disadvantages regarding this insulating liquid, including its high fire hazard and low biodegradability potential. These disadvantages have made it necessary to search for another environmentally friendly ecological insulating liquid alternative to naphthenic oil and which has a high solubility in water and an excellent biodegradability. Different natural esters have already been tested and compared with naphthenic oil [9,11,12,15,19,20]. After a comparison between different mineral and vegetable insulating oils Pukel G. J. et al. [19], from Siemens (Siemens Transformers Austria), noted that esters are more prone to leak breakage than mineral oils. In 2006, D. Martin et al. found that natural esters, in comparison to naphthenic mineral, oil have higher ignition and flash points and that these esters are applicable and adaptable to low voltage transformers [21]. Beroual et al. compared the tendency to electrostatic charge (ECT) of mineral oils to that of vegetable oils; although they noticed some difference, they concluded that this difference did not amount to an obstacle to the use of vegetable oils as insulating and cooling liquid of electrical appliances [13,15]. Other researchers have studied mixtures of mineral oil with vegetable oils for the insulation of power transformers [9,10,12]. M. bin Yahya et al. showed that mixtures of olive oil with naphthenic oil perform better than similar mixtures of sunflower oil [9], they came thereby to the conclusion that, compared to pure naphthenic mineral oil, vegetable oil mixtures performed better in terms of voltage breakdown and that the best ratio of fresh mineral oils and olive oil was 50%. While for sunflower oil it was 75% SO + 25% MO (75% Sunflower Oil + 25% Fresh Mineral Oil). We note that these results reached by these researchers were obtained for a relatively short aging time and almost without taking into account the physico-chemical properties of these oils. In our study, which is mainly concerned with the possible correlation between the ECT and EHD properties of liquid dielectrics, we make use of these same mixture ratios to study the possibility of the existence of a correlation between these unconventional properties et al. the same time to examine the validity of these results during a longer aging time interval.

## 2 Probable diagram of initiation and development of ES and EHD properties leading to breakdowns of forced oil flow transformers

We know that the contact of a liquid (insulating oil) with a solid (insulating paper, metal) is the basis for the formation of an electrical double layer, which is the case existing in power transformers. Depending on the importance of the diffuse layer and especially its thickness and its charge density (which depends on the Debaye length), the forced flow of oil can separate a part of this positively charged layer and transport it, causing a modification of the conditions of electrostatic equilibrium at the solid-liquid interface. A large amount of positive charges separated and carried by the oil flow accumulates in the upper area of the transformer while the rest of these charges are discharged to the oil tank [[27], [28], [29]]. The existence of an alternating field and the creation of a field of electrostatic charges lead to the deformation of the distribution of the electric field, which is more or less similar to what has been reported by other researchers [27,29]. Some researchers assume that the phenomenon of electro-convection can play a negative role: turbulent movement inside the transformer, as well was a positive role: transporting in particular solid waste which can appear with in the oil [25]. According to our hypothetical scenario about the damage to the power transformers, the evolution of the electro-convection phenomenon in the oil cannot play a negative role of any significance compared to its positive role. The oil pumping in the transformer is carried out in a closed circuit, which explains the impossibility of evacuating this solid waste and these gaseous bubbles outside this circuit. For this reason we believe that the appearance of a significant quantity of solid waste in the oil clearly leads either to the damage of the transformer or to the decrease in its lifetime. In any case, we assume that the development of the phenomenon of electro-convection in the oil can contribute to the transport of solid waste (insulating paper, steel) which will accumulate at a certain level inside the transformer and lead to an undesirable increase in its temperature.

**Fig. 1:**
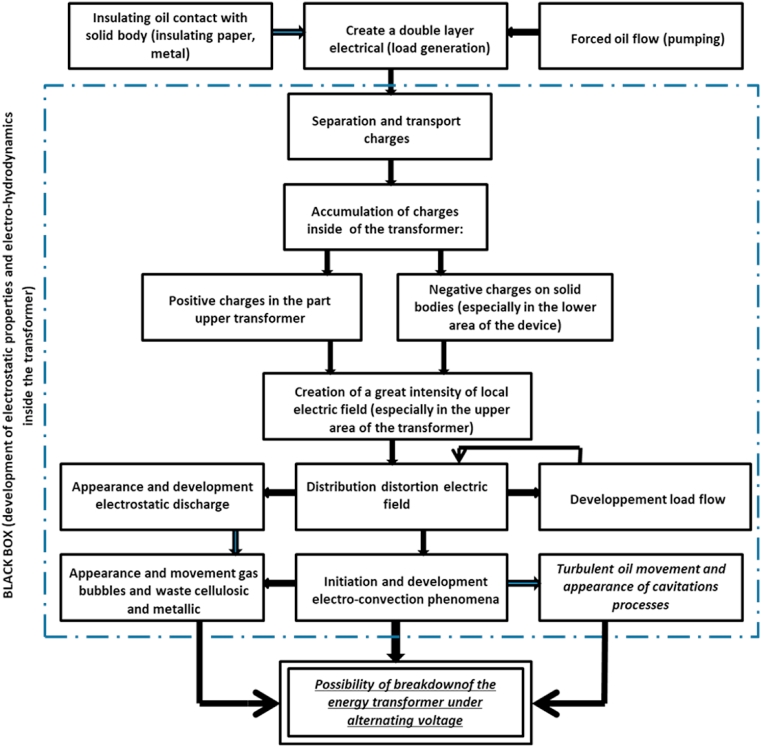
Different probable steps leading to the initiation and development of unconventional properties of insulating oils and subsequently to the breakdown of energy transformers.

As shown in Fig. 1, the distortion of the electric field distribution leads to the development of electrostatic discharges. The development of these discharges and electro-convection gives rise to a turbulent movement in the oil which causes the appearance of cavitation processes, gas bubbles and solid waste (metallic and cellulosic) due to the development of the static electrification phenomena and electro-convection. All of these complicated phenomena can therefore lead to the breakdown of the forced oil flow transformer.

## 3 Devices for studying electrostatic and electro-hydrodynamic properties

Over the past decades, many failures of forced oil flow power transformers have occurred worldwide. These failures, probably caused by static electrification processes, have highlighted the need to develop methods for testing the unconventional properties of transformer oils.

### 3.1 Device for studying electrostatic properties

**Fig. 2:**
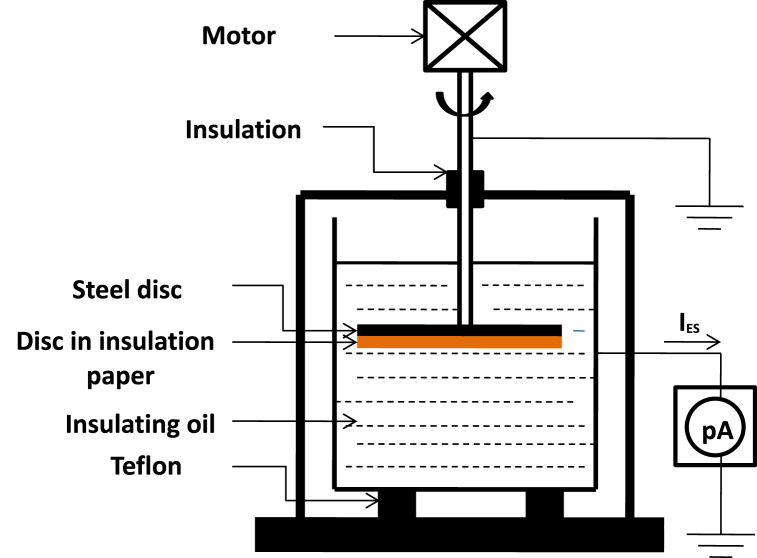
Device for measuring electrostatic charges (CIGRE cell).

The measuring device isolated from any other element (recommended by the members of the CIGRE) consists of a disc rotating in an oil container [26]. The measurement of the electrification currents flowing from the disk to the ground is carried out using a high-precision electrometer. These measured currents are a few pico-amperes. To avoid external disturbances, the measuring device is placed in a Faraday cage (Fig. 2). For the measurements to be repetitive, the measured values fig. 2are taken into consideration only after the first 5 min of rotation of the disc in oil have passed. In our case, the rotating disc consists of two discs of the same materials used in transformers (one in steel and the other in cellulosic paper) and of the same 150 mm diameter glued respectively to each other.

### 3.2 Device for studying electro-hydrodynamic properties

**Fig. 3:**
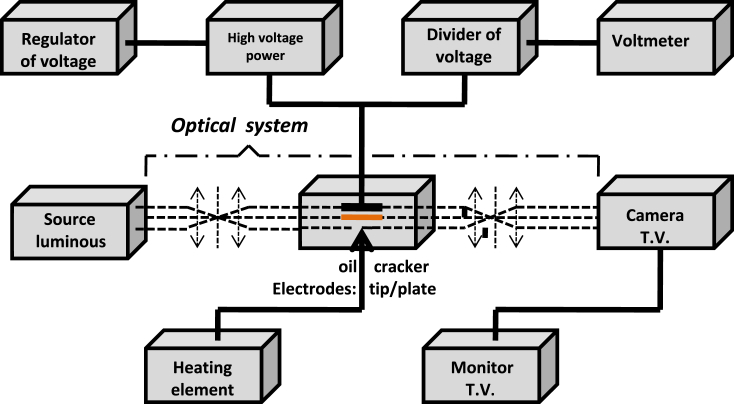
Diagram of the station for studying the electro-hydrodynamic properties of insulating oils.

There are quite a few special methods for studying the initiation and development of electro-convection in liquid dielectrics [5,14,17,18,24,25]. To conduct our studies, we chose a method used by several researchers known as the shadow method [24,25]. It is a method which is based on the use of an optical system for the observation of the movement of the liquid under the effect of the application of a DC or AC voltage. Eventually, the observation of the movement of oil from one electrode to the other is ensured by applying a very small temperature gradient which has practically no effect on the initiation and development of electro-convection phenomena (Fig. 3).

The principle of the measurements of the electro-convection initiating voltage consists in applying an electric voltage to the electrodes fixed in the spark gap containing the oil and in raising this voltage little by little until the observation of the movement of this dielectric liquid. The movement of oil from one electrode to the other is observed on the screen of the TV monitor and recorded by a fast camera (Fig. 3).

## 4 Principles of measurement and preparation of oil samples

### 4.1 Preparation of oil samples

The preparation of oil samples in the laboratory consists of aging artificially in an accelerated manner several samples of naphthenic oil and mixtures of vegetable and naphthenic oils. The samples of aged oils are obtained at a temperature of 95 °C, in the presence of a copper catalyst and in direct contact with air. In this way, oil samples with different degrees of aging are obtained during well-determined time intervals.

### 4.2 Principles of measurement of ECT and EHD properties

Examining this correlation between the non-conventional properties of insulating oils used for the insulation and cooling of energy transformers is based on the study of.

- Electrostatic properties: susceptibility of the oil to static electrification adopted in the ECT literature (electrostatic charging tendency);
- Electro-hydrodynamic properties: flexibility of oil in relation to movement in an electric field which has also been adopted in the EHD literature.

To prove the accuracy of the hypothesis on the existence of a correlation between the ECT and EHD properties of insulating oils (Fig. 1) we measure the quantities characterizing these unconventional properties, i.e. we measure the electrification currents IES and the electro-convection voltages Ui of these oils.

In our study we have carried out the measurements for many samples of different types of mixtures of insulating oils and naphthenic oil, artificially aged under laboratory conditions. The study of the ECT properties of the different oils is as follows: We measure the electrification currents I_ES_ in the rotating disc system shown in Fig. 2 (steel disc on which a cellulosic paper disc is glued). For each oil sample we use a new disk whose purpose is to avoid all the characters that may come from the sample studied previously. During our study we have chosen for the drive of the disc in oil a constant speed ω = 350 rpm. After each measurement, we wash and dry the oil container properly (washing with carbon tetrachloride, rinsing with distilled water, drying at a temperature of 50 °C). For each sample we repeat the measurement of the static electrification current I_ES_ four times and we take the average value of these 4 measurements as the final value.

With regard to the study of EHD properties, we measure the electro-convection initiating voltage Ui in the tip-plate electrode system (Fig. 3) for a.

- Alternating voltage 50 Hz (Ui^∼^);
- Constant voltage and positive polarity of the tip electrode (Ui^+^).
- Constant voltage and negative polarity of the tip electrode (Ui^−^).

In the same way as for I_ES_, we repeat the measurement of Ui four times and we determine the average value of these four measurements and we take it as the final value. During the Ui measurements, we use each oil sample to study its own electrode plate covered with insulating paper. For the tip electrode as well as the oil spark gap, after each measurement they are cleaned and dried, so that they do not contain characteristics or traces from the previous measurement. Between each two measurements, a time interval of 10 min is allowed in order to dissipate gases that may occur during the electric discharge.

## 5 Results and discussion

### 5.1 Correlation study between ECT and EHD

#### 5.1.1 Parametric interpretation

**Fig. 4:**
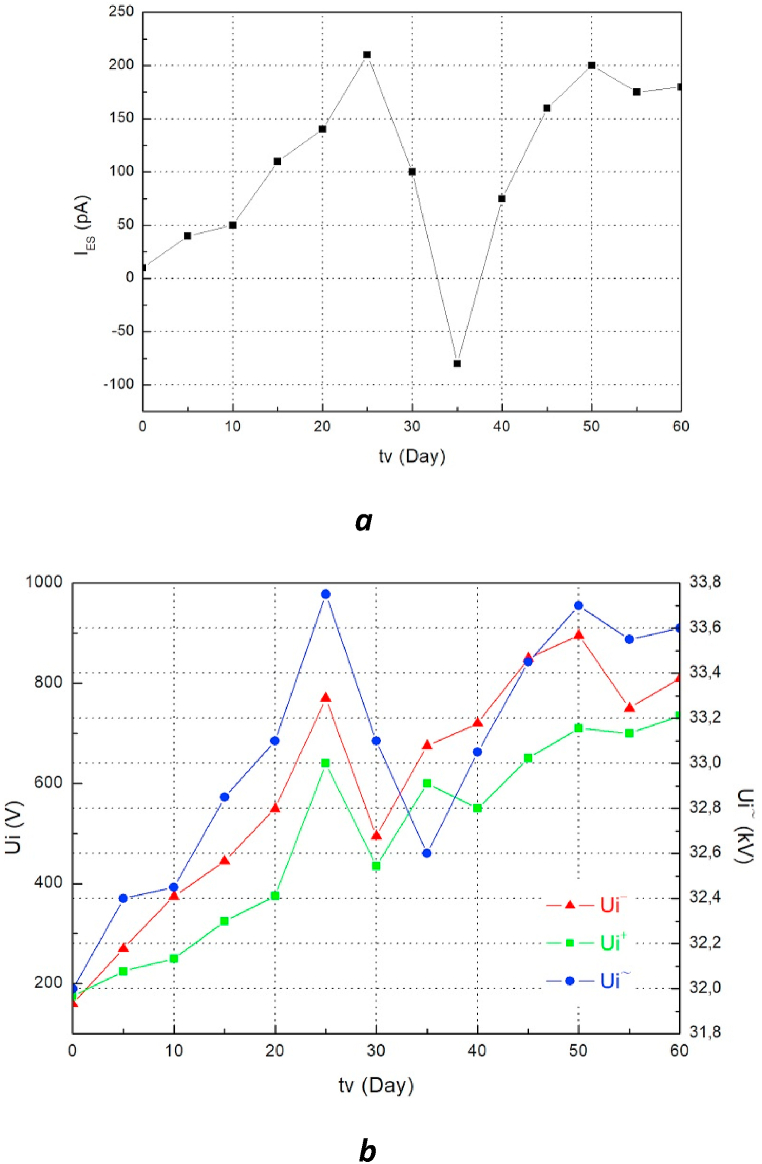
a. Evolution of I_ES_ = f (tv) of the aged naphthenic oil **b.** Evolution of Ui = f (tv) of the aged naphthenic oil.

**Fig. 5:**
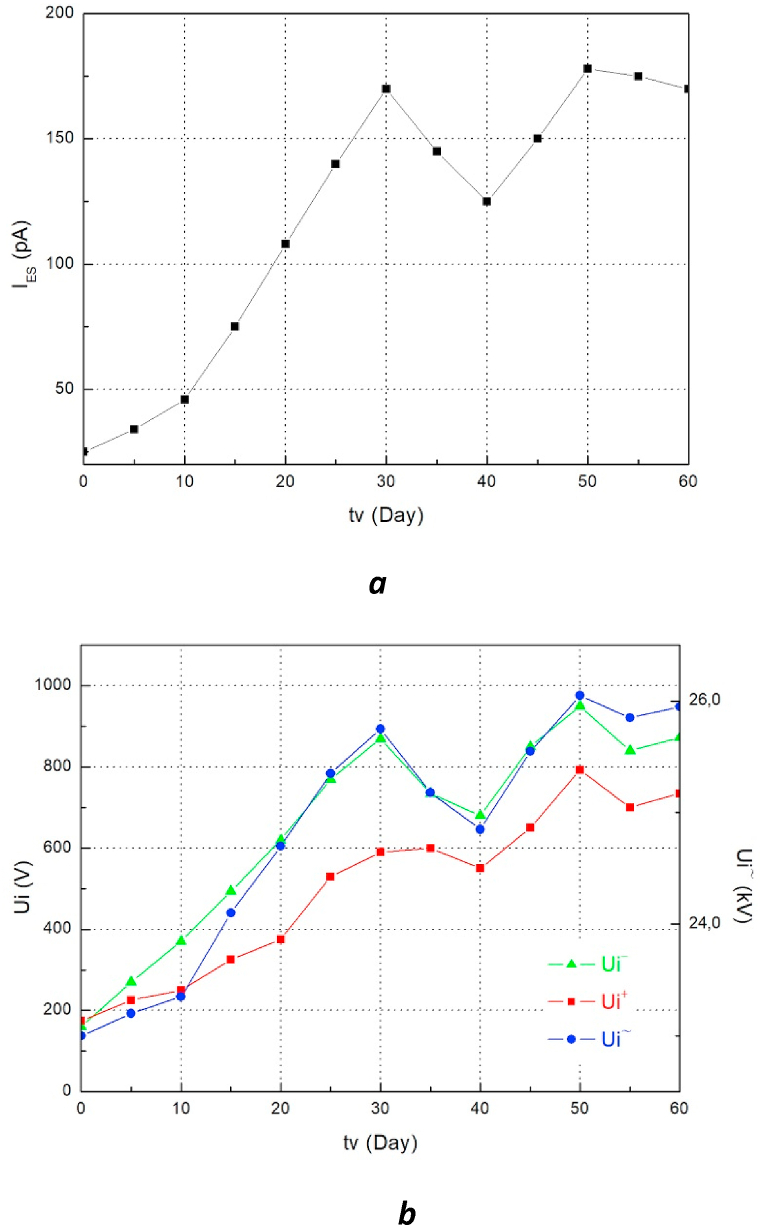
a. Evolution of I_ES_ = f (tv) of the aged oil mixture (50%MO+50%OO) **b.** Evolution of Ui = f (tv) of the aged oil mixture (50%MO+50%OO).

**Fig. 6:**
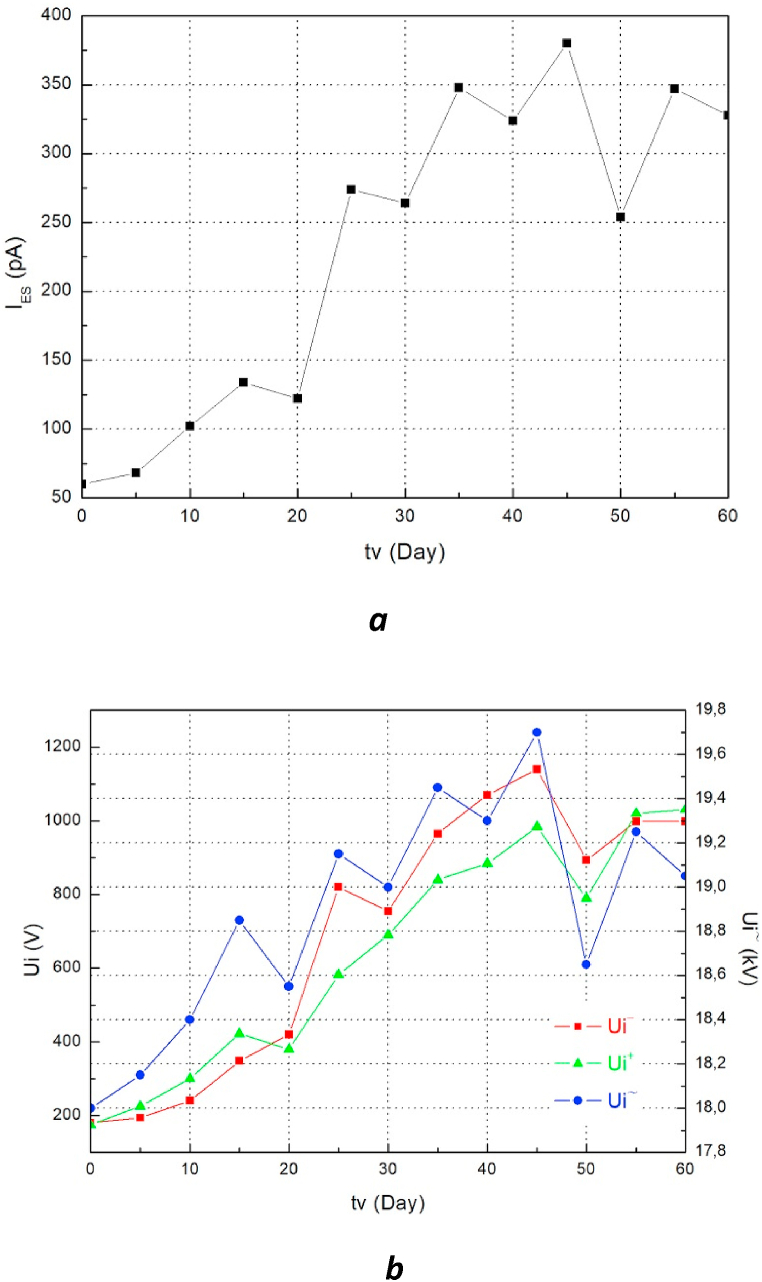
a. Evolution of I_ES_ = f (tv) of the aged oil mixture (75%SO+25%MO) **b.** Evolution of Ui = f (tv) of the aged oil mixture (75%SO+25%MO).

The results of the measurements of naphthenic aged oil samples as well as the different types of mixture are presented in the form of the relationship of the static electrification current I_ES_ (Fig. 4a, Figs. 5a and 6a) and initiating voltage of electro-convection Ui (Figs. 4b, 5b and 6b) as a function of aging time t.

The figures show the characteristic changes of the two measured values (I_ES_ and Ui) during the aging of the oils. We clearly notice that the changes are very similar. The increase in the static electrification current I_ES_ is accompanied by an increase in the electro-convection initiating voltage Ui and the decrease in the current I_ES_ is "reflected" in the graphs Ui = f (tv) also through decreasing values. We can also notice that the extreme values of I_ES_ and Ui appear during the same aging times. We also observe a change in the direction of the I_ES_ current that occurs during the aging cycle of the naphthenic oil which is very interesting. This phenomenon is undoubtedly caused by a change in the sign of electrification of this type of oil during aging.

Apart from the correlation study between the ECT and EHD properties as a function of aging time, it is interesting to report on the possibility of using these mixtures as an alternative to mineral oil. The results obtained show that the charge tendency makes it possible to compare oils with each other, in addition to being a very interesting characteristic for evaluating their electrostatic behavior. These results, presented in the figures (Figs. 4a and 5a), show that the I_ES_ values of the mixture of olive oil with naphthenic oil and those of naphthenic oil alone as a function of aging time don't differ much. Although the I_ES_ value measured for the naphthenic oil changes direction between the 30th and 40th day of aging, which is not the case for the two mixtures, the electro-convection initiating voltage values especially as an alternative for these two insulating liquids are to some degree close to each other. Regarding the values obtained from the I_ES_ of the mixture of sunflower oil with naphthenic oil, we notice that these values are higher in comparison with those obtained for the fresh naphthenic oil. The AC initiating voltage values causing the movement of the insulating mixture (Sunflower SO) are much lower than those of the naphthenic oil. From these results, we can conclude that the mixture of 50% OO + 50% MO can be an alternative to the naphthenic oil used until now for the insulation and cooling of transformers. This finding confirms the results published by M. Bin Yahya et al. who have shown that this oil mixture has better performance and can replace mineral oil for the insulation of energy devices [9]. The characteristic I_ES_ = f (tv) of the mixture of naphthenic oil with sunflower oil in comparison with that of fresh naphthenic oil indicates that this mixture is called into question from the point of view of its capacity to serve as an alternative to mineral oil since it has a great tendency to charge with respect to the latter.

#### 5.1.2 Physical interpretation of results

**Fig. 7:**
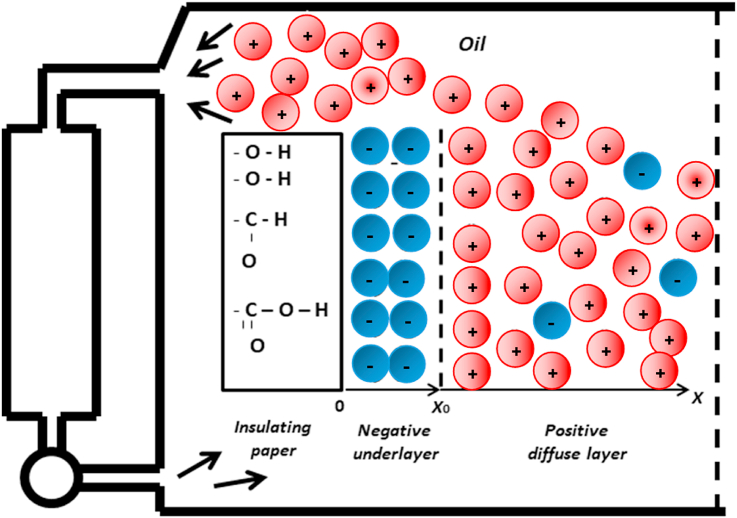
Formation of the electric double layer at the solid-liquid interface.

The contact of a solid with a liquid leads to the formation of a zone in which the intensity of the electric field has a value different from zero. According to Stern (1924), this zone, which goes by the name of the electric double layer, consists of two parts. The first part of this layer consists of adsorbed ions and has a thickness of the order of the size of the ions. The second part is called the diffusion layer which thanks to the thermal movement it is blurred for a certain length in the depth of the liquid. The same phenomenon therefore occurs inside the transformer. The contact of the cellulosic paper with the insulating oil gives rise to the formation of an electrical double layer (Fig. 7).

The latter is formed of ions from the liquid and ions and electrons from the solid bound by electrostatic forces [23]. When the insulating oil is in contact with the insulating paper used in the transformer, the oil becomes positively charged and the paper negatively [1,2]. On the other hand, when the oil is in contact with a metallic body, it can become positively or negatively charged depending on the nature of the solid.

The charge density ρ of the diffusion layer, as shown in Fig. 7, depends on the Debye length δ which determines the width of this layer (Eq. (1)).

(1) $$\delta ={\left(D.\tau \right)}^{1/2}$$

Where: D - molecular diffusion coefficient;

τ - relaxation time, defined as the ratio of the dielectric permittivity ε and the conductivity γ of the liquid (τ = ε/γ).

The distribution of cargo density in oil along the x-axis perpendicular to the boundary surface is described by equation (Eq. (2)) [1]:

(2) $$\rho ={\left(\sigma_{0}/\delta \right)e}^{\left(s-x\right)/\delta }$$

Where: s - surface diameter of the load.

$\sigma_{0}$ - surface charge density.

A particularly important parameter is the conductivity of the liquid, which can vary considerably even in insulating oils. Therefore, it can, in accordance with the relations Eqs. (1) and (2), affect the width of the diffusion layer and the density of the cargo it contains.

Based on this analysis, we can conclude that the studies we have carried out confirm our hypothesis presented in Fig. 1 concerning the existence of a correlation between the electrostatic and electro-hydrodynamic properties of insulating oils since these properties are based on the same theory of the formation of the electric double layer. The physical interpretation of the results obtained is as follows: The forced flow of the oil inside the transformer separates part of the diffuse layer in the form of static charges and transports it where it can accumulate in the upper area of the power device. The accumulation of charges leads to the creation of a significant intensity of the electric field which in turn leads to the initiation and development of electro-convection phenomena. It should be noted that whenever the diffuse layer is wide, charge separation becomes easier.

#### 5.1.3 Static interpretation of results

In the study of the correlation of the two measured values (I_ES_, Ui), a statistical analysis of the results was carried out using the computer program SigmaPlot. In this program, the method of least squares is used to derive the parameters of the regression equation. It consists in determining the coefficients of the equation Eq. (3):

(3) Y = α_0_X_0_ - α_1_X_1_-α_2_X_2_ - …..α_k_X_k_ - B.

Where: *Y* and *k -1* - dependent variables.

*X*_*0*_, *X*_*1*_, *X*_*2*_, …, *X*_*k*_ - independent variables

*α*_*0*_, *α*_*1*_, *α*_*2*_, …, *α*_*k*_ - unknown coefficients.

*B* - Vector of random variables. It is a measure of *Y* deviation from the exact linear relationship between *Y* and *k* independent variables.

In order to find the y-intercept, which is the evaluation of *α*_*0*_, we assume that *X*_*0*_ = 1.

The necessary condition to solve the equation (Eq. (3)), is that the number of observation points is greater than the number of regression coefficients. A particularly simple solution is to use matrix notation. Then the independent variables correspond to the matrix form (Eq. (4)):

(4) $$X=\left[\begin{array}{ccccc} X_{10} & X_{11} & X_{12} & \cdots & X_{1k}\\ X_{20} & X_{21} & X_{22} & \cdots & X_{2k}\\ \vdots & \vdots & \vdots & \cdots & \vdots \\ X_{n0} & X_{n1} & X_{n2} & \cdots & X_{nk} \end{array}\right]$$

and dependent variables and coefficients (Eq. (5))

(5) $$y=\left[\begin{array}{c} y_{1}\\ y_{2}\\ \vdots \\ y_{n} \end{array}\right],\alpha =\left[\begin{array}{c} \alpha_{0}\\ \alpha_{1}\\ \vdots \\ \alpha_{k} \end{array}\right],B=\left[\begin{array}{c} B_{1}\\ B_{2}\\ \vdots \\ B_{k} \end{array}\right]$$

Given the markings entered, the model (Eq. (6)) can be written as follows:

(6) y=Xα–B

According to the method of least squares, the condition for minimizing the sum of the squares of the residuals in the matrix symbolization can be written as follows:

(7) *Ψ* = [Y - Xa]^T^ [Y - Xa]

Condition (Eq. (7)) is fulfilled when the evaluations of the structural parameters are equal to (Eq. (8)):

(8) a = (X ^t^X^−1^) X^T^Y

The evaluation of the variance of the random component B is equal to (Eq. (9)):

(9) $$S^{2}=\frac{1}{n-k-1}\;\left[y^{T}y-y^{T}X\left(X^{T}X^{-1}\right)X^{T}y\right]$$

When calculating $S^{2}$, the matrices calculated for (Eq. (4)) are used.

The average errors of the estimate of the parameter Sa (i = 0,1,2, …, k) are equal to the square root of the terminator at the intersection i - that column of the matrix of variances and covariance which is equal to (Eq. (10)).

(10) $${W^{2}\;(a)=\sigma^{2}\;(X}^{T}X^{-1}$$

Instead of the unknown parameter σ^2^, which is the variance of a random component, its estimated $S^{2}$ is used. The formula used takes the following form (Eq. (11)):

(11) $${W^{2}\;\left(a\right)=S}^{2}{\;(X}^{T}X^{-1}$$

The correlation coefficient noted in the program by R can take the values −1 to +1. If the factor is +1 or −1, we are dealing with a positive or negative linear function relationship. Usually, however, the correlation coefficient will be different from the +1 and −1 threshold values. The value of the coefficient R makes it possible to determine the degree of dependence between the phenomena studied. If the correlation coefficient has approached zero, this means that there is no statistical relationship between the phenomena studied; conversely, if the correlation coefficient has approached +1 or −1, the relationship is very strong.

The results obtained from the statistical analyses leading to the determination of the correlation coefficient for the three types of aged insulating liquids are presented in Table 1.

**Table 1:** Correlation coefficient between static electrification current I_ES_ and electro-convection initiating voltage Ui for different types of aged insulating liquid.

Types of oil	Sample aging time (day)	Correlation coefficient R		
		I_ES_ = f (Ui^−^)	I_ES_ = f (Ui^+^)	I_ES_ = f (Ui^∼^)
Oil MO	from 5 to 60 days	0,925	0,928	0,911
oil mixture 50%MO+50%OO	from 5 to 60 days	0,918	0,908	0,922
oil mixture 75%SO+25%MO	from 5 to 60 days	0,905	0,917	0,915

The values of the correlation coefficients R for the different types of aged oils studied (naphthenic oil as well as for the two other mixtures) are quite high. They show that there are strong correlations between the values of the static electrification current and the values of the electro-convection initiating voltage (I_ES_ and Ui) obtained during our study. The highest correlation coefficient R is obtained for the naphthenic oil followed respectively by that of the mixture 50% MO + 50% OO and 75% SO +25% MO. For a DC initiating voltage, the highest correlation coefficient is obtained for a positive pole of the tip electrode: I_ES_ = f (Ui^+^).

The values of the correlation coefficients R presented in Table 1 vary between 0,905 and 0,928. They must be considered as very high and testify to a very strong correlation between the values tested, characterizing the susceptibility of the oil to static electrification (electrostatic properties) and the tendency of the oil to electro-convection in an electric field (electro-hydrodynamic properties). It is important to note that those correlation coefficients that are by far the highest are obtained for oil.

### 5.2 Physico-chemical properties of insulating liquids

**Fig. 8:**
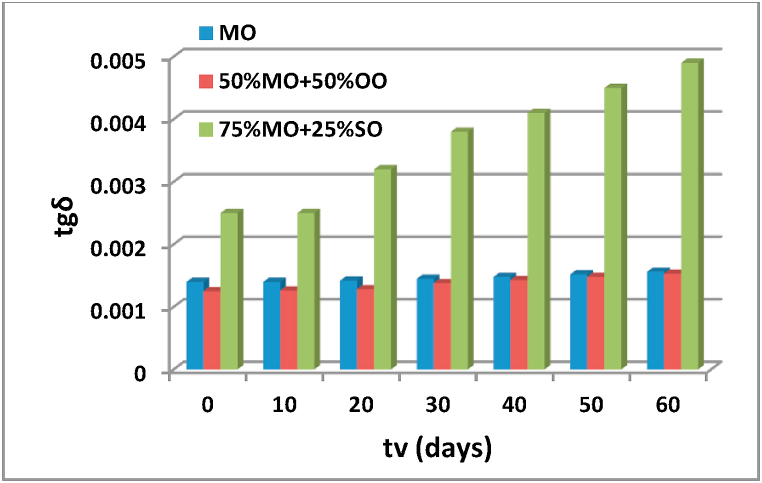
Evolution of the dissipation factor tgδ as a function of the aging time of the different insulating liquids.

**Fig. 9:**
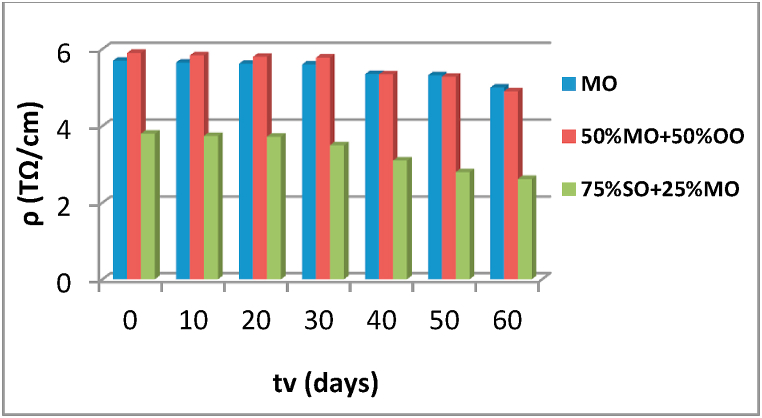
Evolution of resistivity as a function of the aging time of the various insulating liquids.

**Fig. 10:**
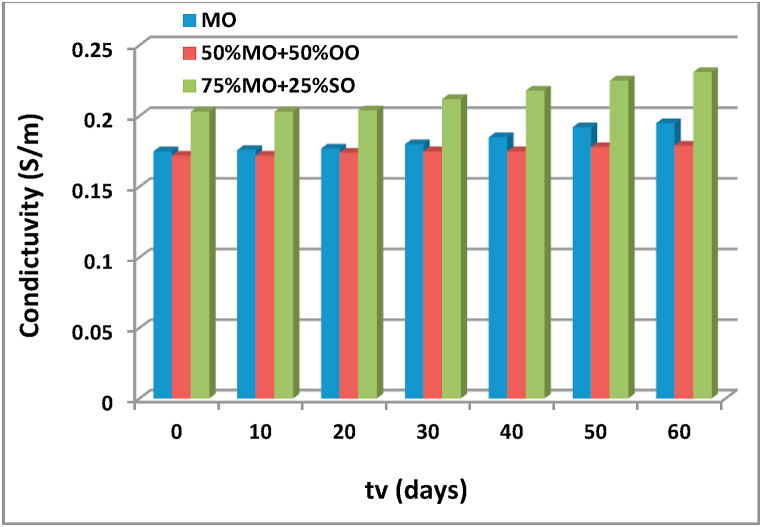
Evolution of the conductivity as a function of the aging time of the different insulating liquids.

**Fig. 11:**
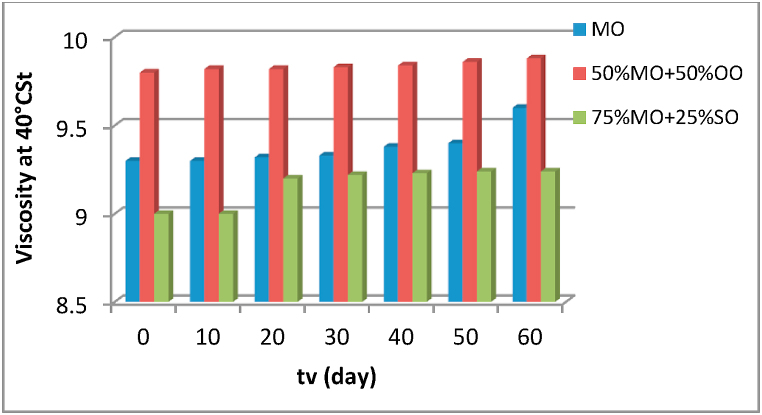
Evolution of the kinematic viscosity as a function of the aging time of the different insulating liquids.

The most important physicochemical properties that characterize the preferred insulating oils for the insulation and cooling of energy transformers are studied and presented in Figs. 8–11. All these properties are measured according to world standards and each measurement is repeated five times for the same aging time. The average value of the five measurements is the value adopted as the final value.

#### 5.2.1 Dissipation factor tgδ

To plot the curves the curves tgδ = f (tv), the determination of the dissipation factor tgδ is carried out according to standard IEC 60247 [9]. We notice from Fig. 8 that the evolution of the dissipation factor which generally signals the electrical quality of the insulating liquid as a function of the aging time for the mixture of 50% olive oil with the same percentage of oil naphthenic does not show much difference. From the beginning of the aging cycle, the tgδ values obtained for the mixture of 75% sunflower oil with 25% mineral oil are higher than those obtained for the two other insulating liquids tested and they increase between the 40th and the 60th day of aging. We find that the mixture of half sunflower oil and half mineral oil is a good insulator since its dissipation factor tgδ does not vary much with aging time and retains values that are not high and are close to that of MO. We also notice that the values of tgδ obtained for the mixture of 75% SO + 25% MO is high compared to that of MO and especially during the interval of aging time between the 30th and the 40th day, which explains that this electrical quality is not acceptable.

#### 5.2.2 Resistivity

The resistivity of the three liquid dielectrics is measured according to IEC 60247 [22]. The resistivity values obtained at 40 °C are presented in Fig. 9. Depending on the aging time, the values obtained for the half mixture of 50% OO + 50% MO do not differ much from that of MO, especially at beginning of the aging cycle. According to these results obtained we find that this mixture of 50% OO and 50% MO is a good insulator, and it has a good capacity to oppose the electric current. Regarding the mixture of 75% SO and 25% MO, the resistivity values for different aging times are lower than that of the other two insulating liquids. They decrease rapidly after the 40th day of aging, which leads to the conclusion that this mixture is not good from a resistivity point of view.

#### 5.2.3 Conductivity

The conductivity values for the different insulators tested are measured using a special IRLAB LDTRP2 type measuring device. The values of conductivity as a function of the aging time obtained are presented in Fig. 10. According to this figure and Figs. 4a, 5a and 6a (I_ES_ = f (tv)), we find that oil aging is accompanied by an increase in the charge tendency which leads to an increase in conductivity. We note that these conductivity values obtained for MO and the 50% OO and 50% MO mixture are lower than that of the 75% SO and 25% MO mixture, which explains why the latter mixture cannot be a good liquid insulator sought for the insulation of power transformers.

#### 5.2.4 Kinematic viscosity

To obtain better insulation, the insulating oil must have a low kinematic viscosity, and this is to allow easy heat transfer inside the transformer. The kinematic viscosity measurements are carried out according to the ISO 3104 standard. Comparison of the values of measured viscosity (Fig. 11) of the two mixtures with that of MO as a function of aging time shows that the mixture of 50% OO with 50% MO is the best despite the fact that its viscosity increases slightly with increasing aging time. For a mixture of 75% SO and 25% MO the values of the kinematic viscosity during the aging cycle are high in comparison with those of mineral oil MO and the half mixture of 50% MO with 50% OO.

The results obtained for the physico-chemical properties of the different insulating liquids studied therefore show that the mixture of 50% OO with 50% MO can be an alternative to naphthenic mineral oil. These results obtained for a half-mixture of mineral and olive oil confirm those published by M. bin Yahya et al. while for a mixture of 75% SO + 25% MO the results are different and remain open to question [9].

## 6 Conclusion

The study carried out on the three types of insulating oil has shown the existence of a clear correlation between the susceptibility of oils to static electrification (electrostatic property characterized by the static electrification current) and their tendencies to electro-convection (electro-hydrodynamic property characterized by the electro-convection initiating voltage). In light of the results of the statistical analysis, this correlation should be considered strong. According to these results, the oil which is very sensitive to static electrification (high static electrification current) is characterized by a weak tendency to move in an electric field (high voltage of initiation of electro-convection) and vice versa. It emerges from these studies that the susceptibility of oils to static electrification and their tendency to electro-convection depend on the same factors, including the type of oil and its degree of aging. The obvious correlation between ECT and EHD of oils results from the fundamental role in the mechanisms of static electrification and electro-convection of the phenomena caused by the presence of the electric double layer at the liquid/solid interface (oil/insulating paper). These phenomena depend on the properties of this double layer whose most important parameter which characterizes it is the width of its diffuse layer (Debye length). The increase in the width of the charge layer facilitates the evacuation of the charges from the interface by the flow of oil (it therefore promotes electrification), while reducing the intensity of the field which occurs there (it weakens the injections of ions, which in turn makes it difficult for the appearance and development of electro-convection).

The measurements of the static electrification currents I_ES_ as a function of the aging time have made it possible to demonstrate that the progressive aging of the oil leads to a remarkable modification of the sensitivity of the oil to electrification. We also notice that in addition to quantitative changes (change in the intensity of charge generation), there may be qualitative changes (change in the sign of the charges generated).

The study of the physicochemical properties of the two oil mixtures (50%MO + 50%OO and 75%SO + 25%MO) such as the dissipation factor tgδ, the conductivity, the resistivity and the viscosity has led us to conclude that a mixture of half naphthenic oil and half olive oil can be an alternative to power transformer oil while a mixture of 75% sunflower oil with 25% mineral oil is open to question.

## Declarations

### Author contribution statement

**Jilani Rouabeh; Walid Nsibi**: Conceived and designed the experiments; Performed the experiments; Analyzed and interpreted the data; Contributed reagents, materials, analysis tools or data; Wrote the paper.

**Lotfi M’barki; Sameh Khmeilia**: Performed the experiments; Analyzed and interpreted the data.

**Brahim Jallouli**: Performed the experiments; Contributed reagents, materials, analysis tools or data.
